# Heating Rate Effect on Gas Permeability and Pore Structure of Mortar under High Temperature

**DOI:** 10.3390/ma15196505

**Published:** 2022-09-20

**Authors:** Wei Chen, Yuehan Liu, Mingquan Sheng, Hejun Zhang, Yue Liang, Frederic Skoczylas

**Affiliations:** 1 School of Civil Engineering, Architecture and Environment, Hubei University of Technology, Wuhan 430068, China; 2 CNRS (Centre National de la Recherche Scientifique), Centrale Lille, UMR9013—LaMcube—Laboratoire de Mécanique Multiphysique et Multiéchelle, Université de Lille, F-59000 Lille, France

**Keywords:** heating rate, porosity, gas permeability, microstructure

## Abstract

This experimental study investigated the effect of heating rate on mortar gas permeability and microstructure. The mortar was heated to three target temperatures (400 °C, 500 °C, and 600 °C) at three heating rates (5 °C/min,10 °C/min, and 15 °C/min). The variations of gas permeability and porosity were measured simultaneously at different confining pressures, and the changes in mortar microstructure were analyzed by NMR and SEM techniques. The results show that the porosity and gas permeability increase with an increase in temperature and heating rate. The gas permeability and porosity continue to decrease as confinement is increased due to a reduction in the pore volume. The microstructure observed by SEM indicates that the high heating rate induces some microcracks at 500 °C and 600 °C. The fractal dimension based on NMR can quantitatively characterize the complexity of the mortar pore structure and shows a quadratic decreasing relationship with gas permeability and porosity.

## 1. Introduction

The effect of high temperature (e.g., fire) not only reduces the mechanical properties of cement-based materials but also has a significant impact on durability [1,2,3,4]. The durability largely depends on the ease with which water and gas can enter into and move through cement-based materials; this is referred to as permeability. The aspect of the microstructure of hardened cement pastes relevant to permeability is the quality of the pore structure and the interface between the cement paste and the aggregate. Determining the water permeability of low porosity and high compactness materials is very difficult, and water saturation has a greater impact on the results. Therefore, the gas permeability is easier to measure and requires less time [5,6,7,8,9].

Transport properties, microstructure, and degradation related to cement-based materials under high temperatures have been largely investigated [10,11,12,13]. Xue et al. found that the maximum water permeability of thermally damaged basalt fiber concrete gradually increased with the increasing temperature, and the permeability gradually decreased during confining pressure unloading [14]. Pei et al. investigated the effect of high temperature at a heating rate of 20 °C/h on the gas permeability of a mortar [15]. Two effects are very clear, an increase in permeability reaching almost two orders of magnitude from 105 °C to 600 °C, and irreversibility in gas permeability caused by the closure of cracks and the crushing (collapse) of the pore. Zhao et al. studied the variation of the microstructure of pre-cracked concrete under different cold methods, the results showed that the surface of specimens cooled in the air after the high temperature at 300 °C became rough. After high-temperature treatment, the crack between the interface of aggregate and cement paste is damaged from the scanning electron microscope image, and the cement compound becomes loose [16]. At 500 °C, after water cooling, the surface of the specimens was severely damaged, and the excessive temperature difference led to cracks in the specimens. Kim investigated the pore structure and development of cracks in cement paste under the effect of high temperature by X-CT, and the results showed that cracks appeared at the edges of the cement paste specimens at 600 °C, and when the temperature continued to increase beyond 900 °C, a crack network started to form [17]. Ye et al. investigated the effects of heating-cooling cycles (heating and cooling rate: 0.5 °C/min) on gas permeability and porosity. It is found that gas permeability decreases during the heating process and increases during the cooling process. The pore volume and gas permeability indicates that microcracks are generated when the temperature exceeds 60 °C [18]. However, most of these studies related to the effects of high temperature, which does not correspond to the different heating rates.

The macroscopic properties of cement-based materials are related to pore structure [19,20,21]. Therefore, the analysis of pore structure plays a crucial role in evaluating the quality of the materials. The pore structure includes porosity, pore size, pore size distribution, and pore morphology. Various techniques and methods have been used to characterize the pore structure, including nitrogen adsorption, scanning electron microscopy (SEM), X-ray computed tomography (X-CT), mercury intrusion porosimetry (MIP), and nuclear magnetic resonance (NMR) [22,23,24,25]. Compared with other methods, NMR non-destructive method can quantify the spatial distribution of pore. Many researchers have studied the pore structure and pore size distribution with exposure time by NMR technology [26,27,28,29]. However, the study of the relationship between permeability and porosity by NMR is still rare.

Fractal geometry is a new branch of mathematics, proposed by the famous French mathematician B.B. Mandelbrot in 1977, mainly for the study of irregularities in nature. In 1985, by using X-ray techniques, Winslow [30] first proposed that cement paste is essentially fractal and proved that fractal theory can quantitatively describe the pore structure of cement paste in terms of complexity and irregularity. In recent years, fractal theory has been increasingly used to study the structure of porous materials, including cementitious materials [31]. The pore structure of cement-based materials, which usually presents extremely complex and self-similar characteristics, is difficult to describe geometrically but can be studied by fractal theory [32]. Therefore, applying fractal theory and calculating the fractal dimension to quantitatively characterize the complexity and irregularity of the pore structure, so as to analyze the relationship between microstructure and macroscopic properties, has gradually become a new research idea for many researchers, which is of great significance for the study of cementitious materials [33,34,35,36,37].

The main goals of this study are to investigate the influence of heating rate on the gas permeability, effective porosity, and microstructure of mortar subjected to high temperature. The mortar specimens were heated from room temperature to the three target temperatures, then naturally cooled to room temperature. The variations of gas permeability and porosity were firstly investigated with a self-designed test device based on the steady-state flow method and gas injection method under varying confining pressure. Secondly, microstructure assessment is performed by scanning electron microscopy (SEM) and nuclear magnetic resonance (NMR), before and after heat treatment, to investigate the main features of microstructure changes. At last, the correlation of fractal dimension based on NMR and gas permeability/porosity of mortar were discussed.

## 2. Experimental Procedure

### 2.1. Materials and Specimens

Mortar with a water-to-cement ratio (W/C) of 0.5 was made with ordinary silicate cement P.O. 42.5 and natural river sand with a fineness modulus of 2.65. The cylindrical mortar specimens of 50 mm × 100 mm were prepared according to the composition shown in Table 1. The specimens were demolded 24 h after casting and maintained in water at 20 °C for 60 d.

### 2.2. Testing Program

#### 2.2.1. Mass Loss Rate

The specimens were raised to target temperatures of 400 °C, 500 °C, and 600 °C in a furnace. Each of these temperatures was achieved by heating rates at 5 °C/min, 10 °C /min, and 15 °C /min. The target temperatures were maintained for 1 h, and then naturally cooled to room temperature. In the previous studies, the saturated specimen was chosen. The highest target temperature of 600 °C led to explosive spalling and consequent damage to the furnace. Therefore, the dry state was considered to be the reference state before the heating. It is obtained after oven-drying at 60 °C until mass stabilization. This temperature was chosen to minimize thermal damage. The mass of mortar, before and after high-temperature damage was weighed with a precision balance to calculate the mass loss of mortar.

#### 2.2.2. Measurements of the Gas Permeability and Porosity

**Figure 1:**
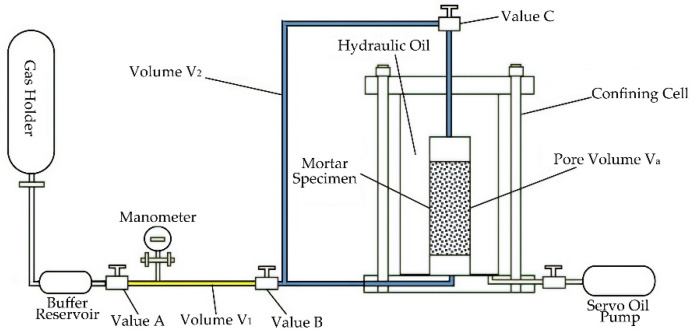
Schematic diagram of the gas permeability and porosity measurement device.

The gas permeability and porosity measurement technique developed in our laboratory for very low permeability (10^−22^ m^2^) have been extensively described [13]. The experimental device mainly consists of a confining cell, a high-precision servo oil pump, and a gas injection panel. The simplified schematic diagram is shown in Figure 1. The mortar specimens were placed in the confining cell at a given hydrostatic pressure Pc, and it is subjected to an inlet gas pressure *P_i_* on one side, and allowed on the other side to freely drain at an atmosphere pressure *P_atm_*. The gas volume flow was obtained using a small pressure decrease ∆*P* during the measuring time ∆t on the specimen upstream side. By assuming of perfect gas state, *P_m_* is the average upstream gas pressure: *P_m_* = *P_i_* − (∆*P*/2). V_r_ is the sum of buffer reservoir volume and pipes volume *V*_1_. *Q_v_* writes:

(1) $$Q_{v}=\frac{V_{r}\Delta P}{P_{m}\Delta t}$$

Gas permeability *K* is given by Darcy’s law, mass conservation, and perfect gas state equation as:

(2) $$K=\frac{2\mu hQ_{v}P_{atm}}{A(P_{m}^{2}-P_{atm}^{2})}$$

where *h* is the sample length, *A* its cross-section, and μ the gas viscosity (2.2 × 10^−5^ Pa.s for argon at 20 °C).

The porosity of the mortar was measured by the gas injection method. The specimen was sealed by confining pressure in the cell. At first, the values B and C were turned off, and value A was open for filling the gas in the tube, the gas pressure was recorded as *P*_1_. In this test, the inlet gas pressure is approximately 2 MPa. When the gas pressure was stable, turning on the values B and C. The gas was injected into the specimen. After the gas was stable again, the gas pressure was recorded as *P*_2_. Before the test, the volume of tubes *V*_1_ and *V*_2_ were measured accurately. According to Boyle’s law, the volume of connected pores *V_a_* and porosity ϕ can be calculated:

(3) $$P_{1}V_{1}=P_{2}(V_{1}+V_{2}+V_{a})$$

(4) $$\Phi =\frac{{}_{V_{a}}}{V}\times 100\%$$

The confining pressure is provided by an oil pump and loaded from 3 MPa, 5 MPa, 10 MPa, 15 MPa to 20 MPa, and finally unloaded to 3 MPa. and the gas permeability and connected porosity under different confining pressures are measured.

#### 2.2.3. Microstructural Analysis

The LF-NMR is used to evaluate the variation of the pore structure of the heat-damaged mortar specimen. Before the test, all specimens were re-saturated so that the internal pores were filled with water. The mass of water in the specimen can be measured by NMR technique and then combined with the density of water, the internal pore volume can be calculated to obtain the internal pore volume so that parameters such as porosity and pore size distribution of porous media materials can be further obtained. Under a static magnetic field, NMR relaxation method can obtain information about the relaxation time. The relaxation time is further divided into two categories, i.e., the longitudinal relaxation time *T*_1_ and the transverse relaxation time *T*_2_, where the transverse relaxation time *T*_2_ is highly sensitive to the pore size, and both satisfy the following equation:

(5) $$\frac{1}{T_{2}}=\rho \frac{S}{V}$$

where *ρ* is the relaxivity strength, *S*/*V* is the surface area to volume ratio of pores.

In scanning electron microscopy (SEM), a small slice (5 mm × 5 mm) was taken from the inside of the temperature-damaged specimen, and the microstructure of the damaged mortar was obtained by SEM after gold spray drying and other treatments.

### 2.3. Determining Fractal Dimension from NMR

In NMR measurements, a *T*_2_ spectrum or *T*_2_ relaxation time distribution are pore volumes corresponding to *T*_2_ value. The calculation model of fractal dimensions by NMR experiment test can be expressed as Equation (6). Taking the logarithm of Equation (6), the calculation of fractal dimension from NMR can be expressed as Equation (7). The relation between both sides of Equation (7) should have a linear correlation. If the correlation is linear, the fractal dimension and maximum pore radius can be calculated by coefficients of the regression equation. The NMR fractal dimension can explain pore distribution.

(6) $$V_{p}={\left(\frac{T_{2}}{T_{2\max}}\right)}^{3-D}$$

(7) $$\log\left(u\right)=\left(3-D\right)\cdot \left(\log\frac{r}{r_{\max}}\right)$$

where *u* is the accumulative volume fraction. *r_max_* is maximum pore radius. *D* is fractal dimension.

## 3. Results and Discussion

### 3.1. Effect of Heating Rate on Mass Loss

**Figure 2:**
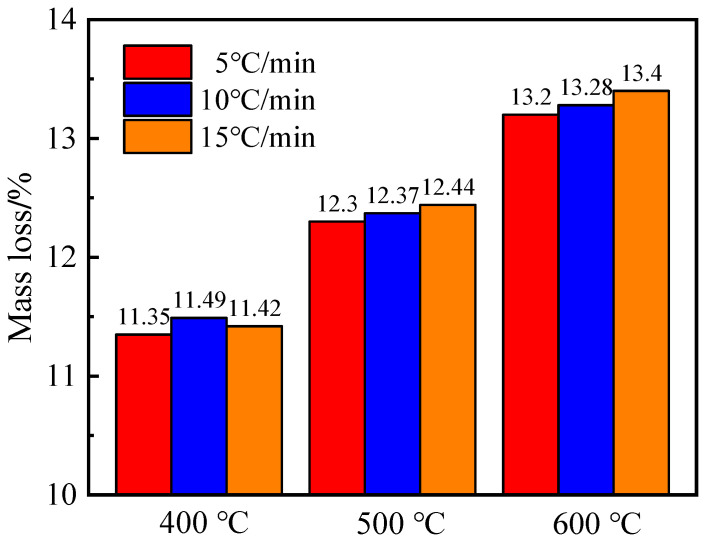
Effect of heating rate on the mass loss of mortar.

As shown in Figure 2, the variation of mass loss of mortar was similar at the different high temperatures. The mass loss increased from 5 °C/min to 15 °C/min. The different phases in a mortar decompose during different temperature ranges when it is subjected to heating. Around 100 °C, free water was released; above 200 °C, the bound water began to lose; C-S-H decomposed from 105 °C and up to 400 °C; portlandite Ca(OH)_2_ released its water molecule from 400 Ca(OH)_2_ to 600 °C. At elevated temperatures, the increase in mass loss can be caused by the decomposition of different phases. The evaporated water transforms into steam and causes pore pressure. When the pressure overcomes the tensile strength of mortar, microcracks begin to develop in the weakened zone such as the interfacial transition zone (ITZ). This is more significant at higher temperatures. The mass loss at a given heating rate was higher above 400 °C. This was similar to the variation of mass loss of mortar and concrete at high temperatures studied by Li [4] and Chen [12].

### 3.2. Effect of Heating Rate on Gas Permeability and Porosity

#### 3.2.1. Initial Gas Permeability and Initial Porosity

In order to better analyze the effect of the heating rate, the initial gas permeability and initial porosity of the mortar under 3 MPa confining pressure are studied firstly.

**Figure 3:**
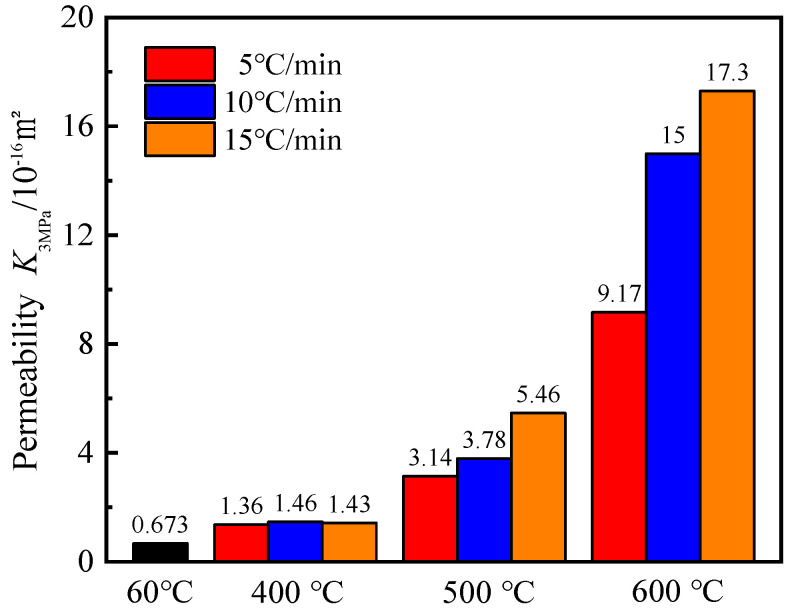
Variations in initial permeability with temperature and heating rate (Inset: permeability with respect to reference temperature 60 °C).

As shown in Figure 3, the mortar gas permeability due to various high temperatures increased by a maximum of two orders of magnitude compared to the reference group (60 °C). The initial gas permeability of the mortar gradually increased with increasing temperature regardless of the heating rate. The effect of the heating rate on the initial gas permeability was more significant at 500 °C and 600 °C. Compared with the reference group, it increased by 13.62, 22.29, and 25.71 times when the mortar was heated to 600 °C at three heating rates. At 400 °C, the gas permeability is similar, but the effect of heating rates is not evident. It can be further concluded that the higher temperature and the faster the heating rate, the greater the effect on the mortar gas permeability.

The mortar changes its own pore structure characteristics after different high-temperature, and its permeability changes similarly. At high temperatures, the dissipation of free and bound water and the decomposition of hydration products inside the mortar lead to an increase in pore size and microcracking [15]. The different thermal expansion coefficients between cementitious materials and aggregates, and the material anisotropy cause cracks at the ITZ. Because of the high-temperature gradient of mortar at a higher heating rate, the expansion rate is different, resulting in cracks in the mortar. Therefore, the gas permeability increased significantly with the higher heating rate when compared to a low heating rate for an identical target temperature.

**Figure 4:**
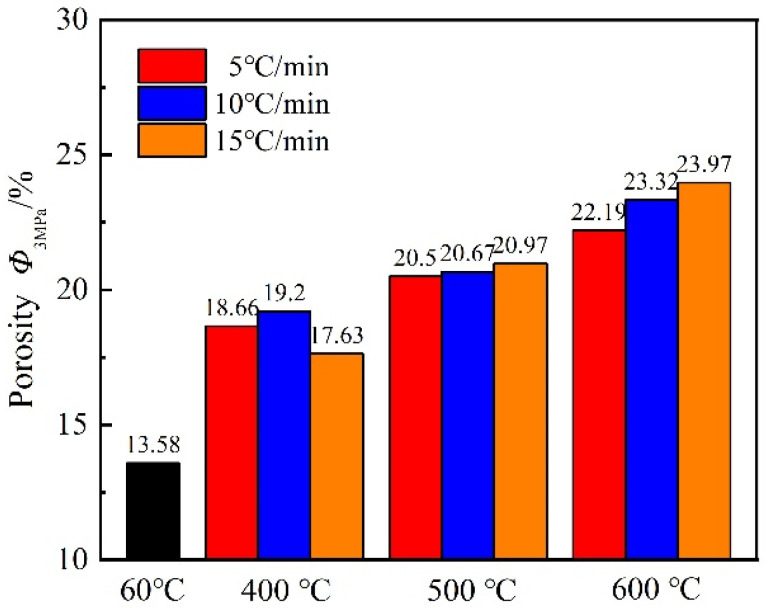
Variations in initial porosity of mortar with temperature and heating rate (Inset: porosity with respect to reference temperature 60 °C).

Porosity is a crucial factor that causes the change in gas permeability. The porosity increases and the mortar gas permeability increases consequently. Figure 4 shows that the changes in mortar porosity with an increase in the temperature and heating rate are similar to the changes in gas permeability. At the same heating rate, the higher the target temperature, the greater the porosity of the mortar. When the mortar was heated at 5 °C/min to target temperatures, the initial porosity of the mortar increased by 1.37, 1.51, and 1.63 times, respectively, when compared with the reference group. At 500 °C versus 600 °C, the faster the heating rate, the greater the porosity. When the mortar was heated to 600 °C at three heating rates, the initial porosity of the mortar increased by 1.63, 1.71, and 1.77 times, respectively, compared with the reference group. At the same target temperature, the difference in porosity was not significant with three heating rates.

#### 3.2.2. Effect of Confinement on Gas Permeability and Porosity

**Figure 5:**
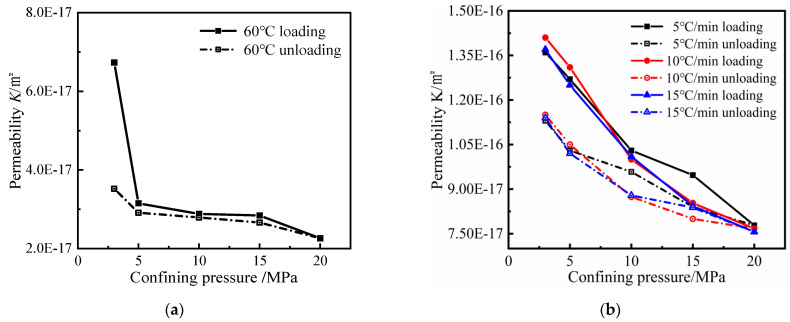

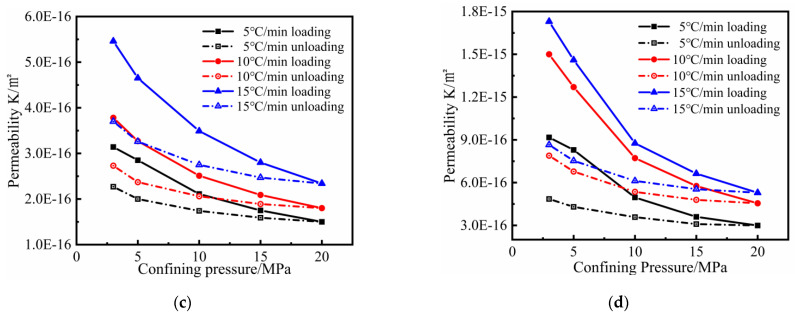
Variation in gas permeability under confining pressure loading and unloading: (**a**) 60 °C; (**b**) 400 °C; (**c**) 500 °C; (**d**) 600 °C.

As for the mechanical behavior, there is the microcrack closure phase under confining pressure. In Figure 5, the effect of confining pressure on gas permeability is very significant, and the gas permeability gradually decreases with the increase in confining pressure whatever the heating rate. The damage of hydrates causes the matrix to become less resistant, and it can be assumed that the effect of confining pressure is more important. The change in gas permeability under low confining pressure is more significant compared with that under high confining pressure, and the change in gas permeability under high confining pressure is slower, which indicates that there is a threshold value between 15MPa–20MPa in the confining pressure on gas permeability.

When the confining pressure was increased from 3 MPa to 5 MPa, the gas permeability of all heated mortars was mainly reduced by about 10%. When the confining pressure was increased to 10 MPa, the mortar gas permeability after 600 °C treatment was reduced by about 40% compared with that at 5 MPa, and by about 20–30% at 400 °C and 500 °C. When the confining pressure continues to increase to 20 MPa, the mortar gas permeability after 600 °C treatment is reduced by about 20% compared with that at 10 MPa, and by about 10–20% at 400 °C and 500 °C. It can be found that the mortar gas permeability after high-temperature exposure has a high sensitivity to the confining pressure. Therefore, it can conclude that there is a sensitive zone of 3–10 MPa and an insensitive zone of 10–20 MPa. At 500 °C and 600 °C, the effect of heating rate is more significant, i.e., the faster the heating rate, the greater the effect of confining pressure on mortar gas permeability.

The changes in the internal pore structure characteristics of mortar by confining pressure lead to a variation in the gas permeability of mortar. After the heated at different heating rates, a large number of pores and cracks are generated inside the mortar, and the increase in the confining pressure leads to the closure of the cracks and the crushing of the pores progressively, while the solid matrix may also fail due to C-S-H decomposition. The gas permeability decreases more obviously at lower confining pressure because some larger pores and cracks inside the mortar are easily affected by the confining pressure and change, so the change is higher at low confining pressure. When the confining pressure continues to increase, the change in gas permeability is relatively small because the pore structures such as large pores and cracks inside the mortar have been closed and only some very small pore structures have been changed, so the change in gas permeability of the mortar is slow at high confining pressure.

In the confining pressure unloading phase, the gas permeability becomes almost irreversible. It gradually increases, but cannot recover the value in the loading phase. The higher the target temperature and the faster the heating rate, the more significant the irreversibility. This is because the high-temperature damage has an impact on the elastic-plastic deformation of mortar, the higher the heating temperature and the faster the heating rate, the less the elastic deformation of mortar occurs, and the plastic deformation is more obvious. It is important that the gas permeability at 3 MPa in the unloading phase is very close to that at 10 MPa in the loading phase.

**Figure 6:**
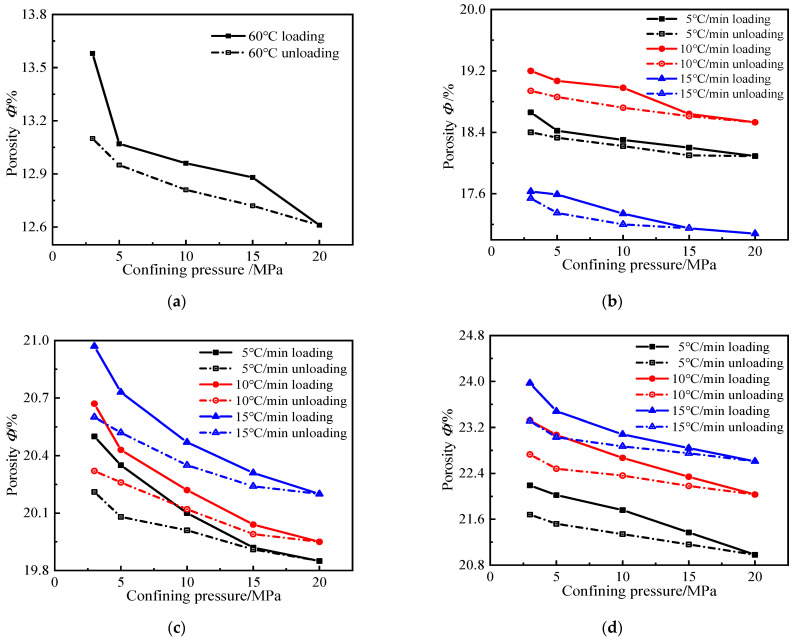
Variation in porosity under confining pressure loading and unloading: (**a**) 60 °C; (**b**) 400 °C; (**c**) 500 °C; (**d**) 600 °C.

**Figure 7:**
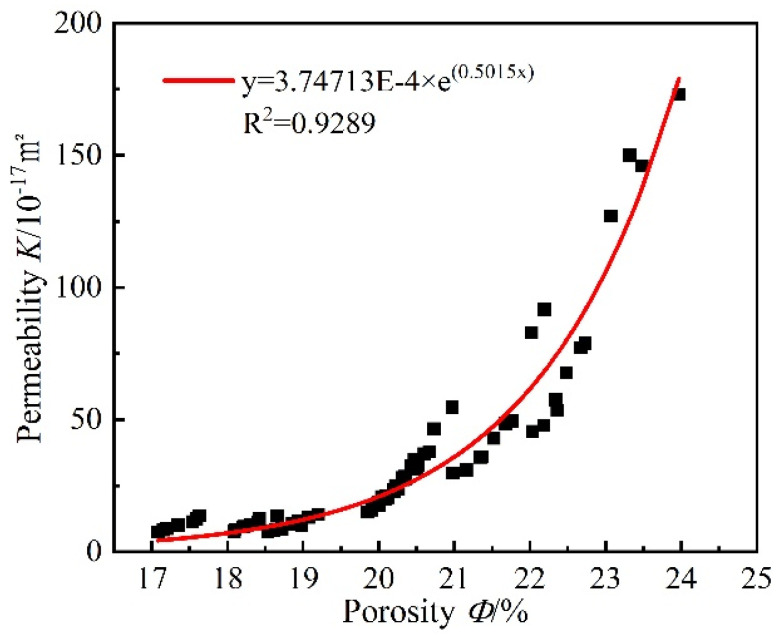
Relationships between porosity and gas permeability (Black square: experimental data).

In Figure 6, it can be seen that the variation of mortar porosity with confining pressure is basically similar to that of gas permeability. The mortar porosity after different high-temperature heats gradually decreases with the increase in the confining pressure, which is because the increase in the confining pressure induces a contraction of the mortar specimen, and the internal pore structure becomes smaller. Additionally, it can be found that there is a threshold value of confining pressure on mortar porosity at a lower temperature (60 °C) and high temperatures. The change in mortar porosity is also more significant at low confining pressure and insignificant at high confining pressure. As in the case of the gas permeability, the porosity of all heated mortar becomes very sensitive to confining pressure and is irreversible during the unloading phase. The change in mortar permeability is closely related to the change in the internal pore structure. Although the difference in mortar porosity during the confining pressure loading and unloading phase is not large, the gas permeability differs greatly, which also implies that the gas permeability is more sensitive to the opening and closing of cracks inside the mortar and the crushing of pores. The sensitivity of porosity to the confining pressure is small compared to gas permeability, but the irreversibility of porosity confirms the irreversible closure of some cracks and crushing of pores. It also indicates that the variation in permeability is closely related to porosity regardless of the type of material used (i.e., treated or untreated) and the level of confining pressure (loaded or unloaded). Figure 7, from which a good exponential fit R^2^ = 0.9289 is determined, describes the gas permeability as a function of porosity.

### 3.3. Pore Structure of Mortar

#### 3.3.1. Image Analysis

**Figure 8:**
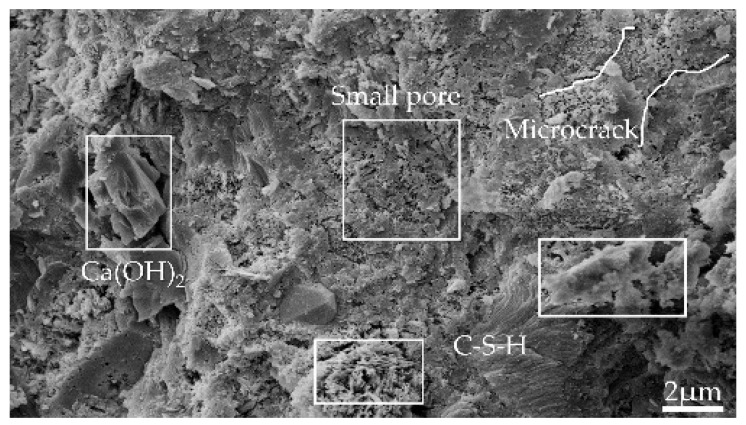
Microtopography and pore structure images of reference group mortar (60 °C).

**Figure 9:**
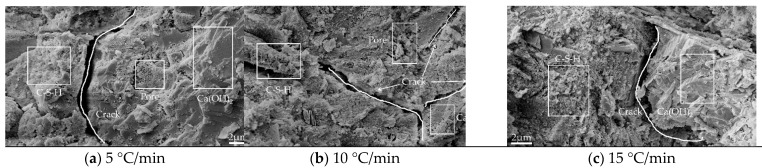
Microtopography and pore structure images of mortar heated (400 °C): (**a**) 5 °C/min; (**b**) 10 °C/min; (**c**) 15 °C/min.

**Figure 10:**
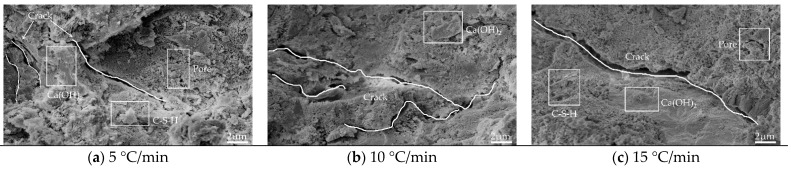
Microtopography and pore structure images of mortar heated (500 °C): (**a**) 5 °C/min; (**b**) 10 °C/min; (**c**) 15 °C/min.

**Figure 11:**
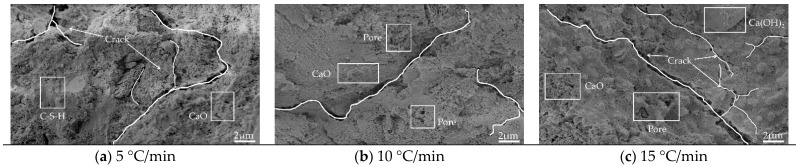
Microtopography and pore structure images of mortar heated (600 °C): (**a**) 5 °C/min; (**b**) 10 °C/min; (**c**) 15 °C/min.

Figure 8, Figure 9, Figure 10 and Figure 11 show the SEM images of the mortar microstructure after different high-temperature damage at a magnification of 5000 times. It can be seen that at 60 °C, the microstructure of the mortar is relatively dense, mainly consisting of hexagonal or layered Ca(OH)_2_ and flocculated C-S-H, with a small amount of initial tiny pores as well as microcracks inside. With the increase in heating temperature, the microstructure of mortar changed significantly regardless of the heating rate. At 5 °C/min, for example, at 400 °C, a small amount of Ca(OH)_2_ decomposes, and the C-S-H tarts dehydrate, with little change in microstructure, but cracks start to appear inside. When the temperature was heated to 500 °C, the dense structure of the mortar began to become loose, and the shape changed from hexagonal and flocculent to porous flakes, and a large number of holes and independent cracks were produced inside the mortar, which was mainly due to the dehydration and thermal decomposition of Ca(OH)_2_ and C-S-H at high temperature and the high heating temperature, due to the different expansion coefficients. When the temperature continues to be heated to 600 °C, it can be seen that the mortar becomes looser inside, Ca(OH)_2_ has been dehydrated and decomposed to produce CaO in large amounts, and no longer presents a complete hexagonal or laminar shape, but presents a sponge shape, etc., and a large number of cracks are produced inside and the cracks begin to interconnect. At 500 °C and 600 °C, with the increase in the heating rate, the internal hydration products of mortar became fragile and rough. The number of microcracks propagated and expanded, and the cracks began to connect to each other.

From the above analysis, it can be concluded that the effect of heating temperature on the microstructure of mortar is more significant than that of heating rate. In the case of low temperature (400 °C), the effect of heating rate on mortar microstructure is not obvious, and with the increase in temperature, the effect of heating rate starts to take effect, i.e., the higher the heating temperature and the faster the heating rate (15 °C/min at 600 °C) the greater the effect on mortar microstructure. Under the effect of different high temperatures, the mortar microstructure mainly showed that the hydration products of mortar started to be decomposed by heat, and the internal structure became porous and loose and produced a large number of cracks, but on the macroscopic level, it mainly showed that the mortar gas permeability increased.

#### 3.3.2. NMR *T*_2_ Spectra and Fractal Analysis

**Figure 12:**
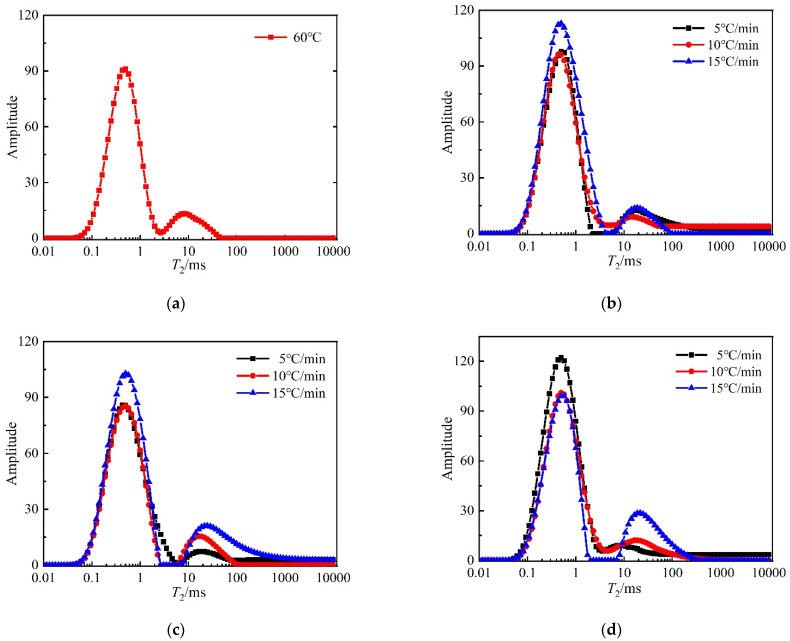
NMR *T*_2_ relaxation time distributions of mortar: (**a**) 60 °C; (**b**) 400 °C; (**c**) 500 °C; (**d**) 600 °C.

The *T*_2_ relaxation time distributions for mortar are shown in Figure 12. The *T*_2_ spectrum is positively correlated with the amount of pore water content of concrete, i.e., the change in *T*_2_ spectrum distribution can reflect the variation of the pore volume of concrete material. In the *T*_2_ spectrum distribution, the horizontal *x*-axis represents the relaxation time, which is proportional to the pore size, the longer the relaxation time, the larger the pore size, and vice versa, the smaller the pore size. The variation of the area of the *T*_2_ spectrum reflects the variation of the pore volume; the larger the area, the larger the pore volume in the area corresponding to the pore diameter, and vice versa [17].

From Figure 12, it can be seen that the internal pore structure of the mortar after high-temperature treatment is changed compared to the reference group. Firstly, there are two peaks in the *T*_2_ spectra of all mortars, and the primary peak is larger than the secondary peak, and the distribution of the primary peak occupies three orders of magnitude in all cases. The range relaxation time corresponding to the primary peak is shorter, then the primary peak represents the change in micro and small pores, while the second peak represents the change in medium and large pores. As the temperature increases and the heating rate increases the main peak shifts to the right and the relaxation time becomes longer, indicating that many micropores are transformed into medium pores during the heating process, etc. The secondary peak also gradually shifts to the right, indicating that some medium pores evolve into large pores. It can also be seen that the secondary peak amplitude gradually increases with the increase in the heating rate at 500 °C and 600 °C, which indicates that the change in the heating rate at these two temperatures has a more significant effect on the medium and large pores inside the mortar.

**Figure 13:**
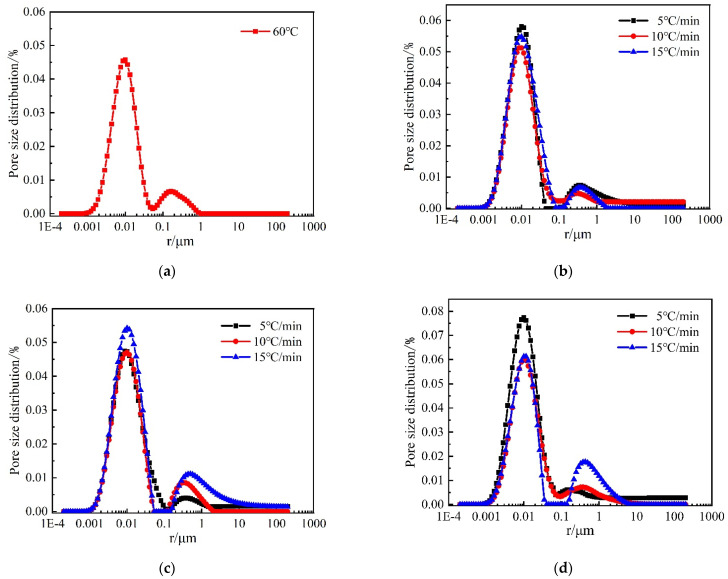
Pore size distribution of mortar: (**a**) 60 °C; (**b**) 400 °C; (**c**) 500 °C; (**d**) 600 °C.

Figure 13 describes the changes in the pore size distribution of mortar after different high-temperature treatments. It can be seen that the pore size distribution plots are basically similar to the *T*_2_ spectra, and there are two peaks in the pore size distribution plots of mortar after different temperature damage, and the primary peaks are larger than the secondary peaks. The pore size distribution of all the mortars after heating is mainly in the range of 0.001~100 μm, and all of them have the largest amplitude of the main peak, indicating that the pore size distribution is the largest in the range of 0.001~0.1 μm. It can be found that the pore size distribution gradually moves to the right with the increase in temperature at the same heating rate, indicating that the pore size inside the mortar gradually increases during the heating process. At the same temperature, with the increase in heating rate, the pore size distribution of mortar gradually increases. At 500 °C, the change in both primary and secondary peaks with heating rate is more obvious, and at 600 °C, the change in secondary peaks with heating rate is more obvious. This further indicates that the higher the heating temperature and the faster the heating rate, the more significant the effect on the pore structure of the mortar.

Based on the mortar pore size distribution in Figure 13, the linear regression equation of pore size and pore volume is established based on Equation (7), and the fractal dimension D of the mortar can be obtained by recalculating the coefficients of the obtained linear regression equation are roughly the same.

**Figure 14:**
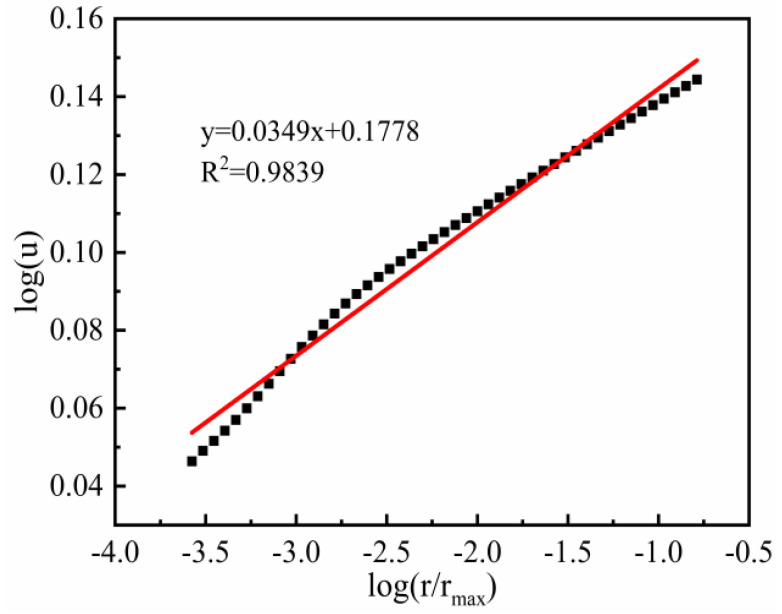
Plot of log (u) versus log (r/r_max_) of pore sizes larger than 0.05 μm of mortar (Black square: NMR test data).

Figure 14 shows the fractal dimension fit for medium and large pores (pore size >0.05 μm) of mortar, and the correlation coefficient is 0.9839, which is a good fit and shows a strong correlation, indicating that the fractal characteristics of medium and large pores are more obvious after high-temperature damage after reducing the pore size range, and these pores play a major role in the macroscopic properties of mortar.

**Figure 15:**
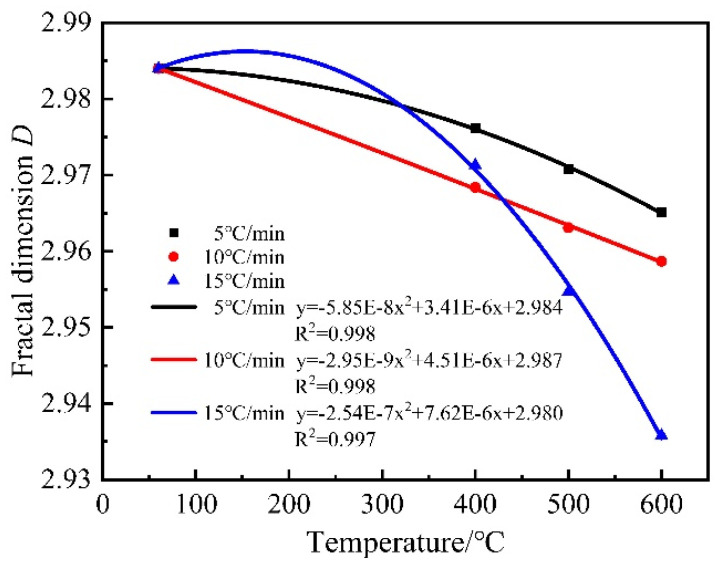
Relationship between fractal dimension and temperature.

**Figure 16:**
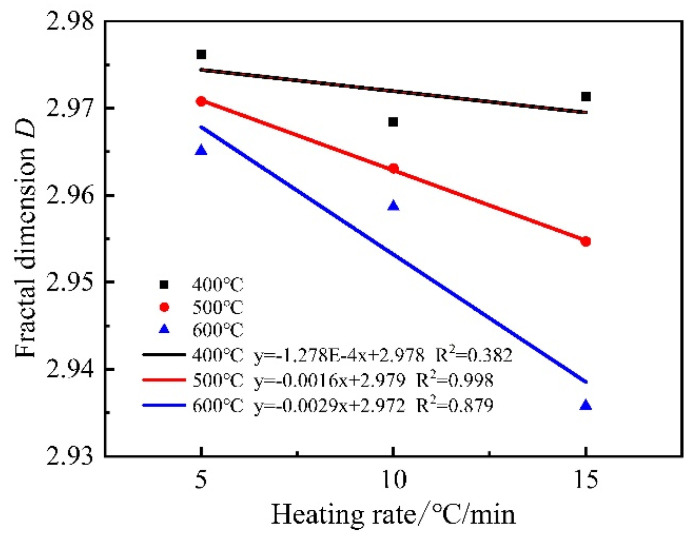
Relationship between fractal dimension and heating rate.

From Figure 15 and Figure 16, it can be seen that the fractal dimension of mortar decreases gradually with the increase in heating temperature at the same heating rate. The fractal dimension of mortar decreases gradually with the increase in the heating rate at 500 °C and 600 °C. The fractal dimension of mortar is maximum at 60 °C and minimum at 600 °C (15 °C/min). This is because, at 60 °C, the pores inside the mortar consisted of many micropores with a complex pore distribution and large fractal dimension, and after the high-temperature treatment, many micropores inside the mortar evolved into medium and large pores, and the pore structure became single, and the fractal dimension was small. This indicates that the fractal dimension of mortar is related to the size of pores, when the number of micropores is relatively large, the distribution density of internal pores is tighter and the pores are intricate and complex, so the fractal dimension is larger, when some pores gradually evolve into medium and large pores, the distribution density of pores is sparse leading to the decrease in fractal dimension. At the same time, the fractal dimension shows a good quadratic function with temperature and a primary function with temperature rise rate.

**Figure 17:**
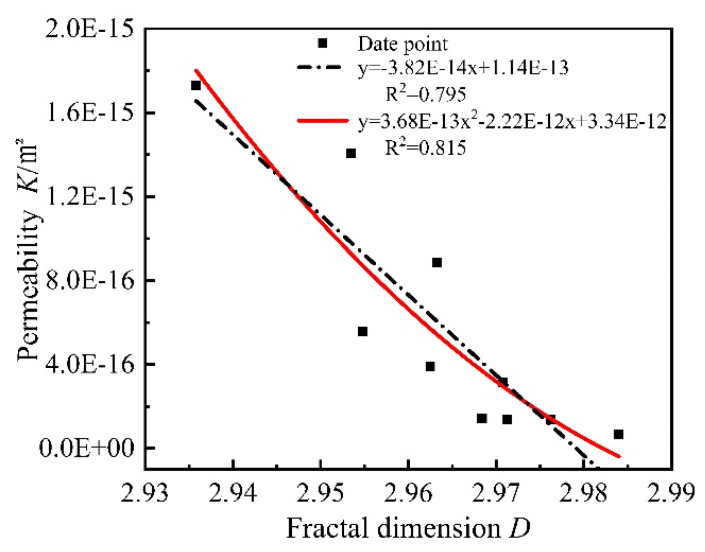
Relationship between permeability and fractal dimension.

**Figure 18:**
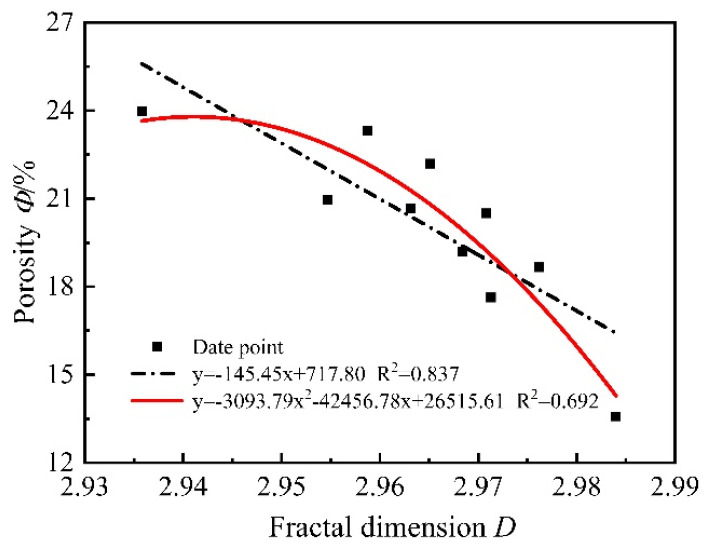
Relationship between porosity and fractal dimension.

In Figure 17, the initial gas permeability of all mortars was fitted with the fractal dimension, and it was found that the polynomial fit was the best among all the fitted relationships, and the fractal dimension showed a negative correlation with the gas permeability, and the best mathematical relationship model was a quadratic function. After high-temperature damage, many small pores in the mortar evolve into large pores, the gas permeability increases, and the fractal dimension of the corresponding pores decreases. It can be said that the percolation path extended leading to a decrease in gas permeability. From Figure 18, the initial porosity of all mortars was fitted with a fractal dimension, and it was found that the polynomial fit was the best among all fitted relationships, the fractal dimension showed a negative correlation with the initial porosity, and the best mathematical relationship model was a quadratic function. This indicates that the larger the fractal dimension is, the more complex the pore distribution is and the smaller the porosity is. The main reason is when the fractal dimension is larger, the pores inside the mortar are mainly small and medium pores, the distribution of different types of pores in the limited space is intricate and complex, the internal pore structure is denser, and the porosity measured by the gas method is smaller. When the mortar is damaged by high temperature, many pores evolve into large pores and the pores pass through each other, the distribution of pore structure inside the mortar is simple, and the porosity increases.

## 4. Conclusions

This experimental study investigates the effect of heating rate on the variations in the gas permeability and microstructure. Three heating rates 5 °C/min, 10 °C/min, and 15 °C/min were selected. The gas permeability and porosity were measured by using the gas injection method, and the microstructure was analyzed with an SEM and NMR. Fractal characteristics of mortar were studied by using NMR analyses. The specific conclusions are as follows:

(1) The initial gas permeability increased by two orders of magnitude and the initial porosity increased by 1.77 times in the mortar after it heated compared with the reference group (60 °C). The higher the target temperature the faster the heating rate has a greater effect on the gas permeability and porosity (i.e., heating at 15 °C/min to 600 °C). The microstructure analysis showed that the variations contribute to an increase in pore size and microcrack generation.
(2) The gas permeability measurement under varying confining pressure displays a different behavior during the loading and unloading phase. All heated mortar showed significant pore volume reduction, which is not recovered after unloading due to the creation of microcracks at the sand/cement paste interface by heat treatment. These microcracks had varying width and tortuosity; therefore, they provide more gas passage at low confinement, and partially closed irreversibly at higher confining pressure. Compared with porosity, gas permeability is more sensitive to confining pressure, and the irreversibility of porosity confirms the irreversible closure of cracks and irreversible collapse of pores during the loading and unloading phase.
(3) Thermal treatment at elevated temperatures may change the microstructure of mortar. The results obtained from SEM showed more micro gaps and microcracks in the mortar with the increased temperatures and heating rates. It confirmed the increase in connectivity of pore and microcrack with increasing temperature.
(4) NMR relaxation is an efficient tool for non-destructively and quantitatively evaluating the pore structure of cement-based materials. Based on NMR experiment data, there is a slight decrease in the fractal dimension of pore volume with the increased temperatures and heating rates. Fractal dimensions calculated from the *T*_2_ spectrum show negative correlation relationships with the gas permeability and porosity.

## Figures and Tables

**Table 1:** Mortar composition.

W/C	Cement (kg/m^3^)	Sand (kg/m^3^)	Water (kg/m^3^)
0.5	450	1350	225

## Data Availability

The data presented in this study are available on request from the corresponding author.
